# MicroRNA-299a-5p Protects against Spinal Cord Injury through Activating AMPK Pathway

**DOI:** 10.1155/2022/8659587

**Published:** 2022-05-13

**Authors:** Zong-Ze Zhang, Shu-Yue Xian, Chong Bao, Feng Chen

**Affiliations:** Department of Anesthesiology, Zhongnan Hospital of Wuhan University, China

## Abstract

**Objective:**

Inflammation and oxidative stress are implicated in the pathogenesis of spinal cord injury (SCI). The present study is aimed at investigating the function and molecular basis of microRNA-299a-5p (miR-299a-5p) during SCI in mice.

**Methods:**

Mice were exposed to SCI surgery and then intrathecally injected with the agomir, antagomir, or matched negative controls of miR-299a-5p to overexpress or silence miR-299a-5p. To inhibit AMP-activated protein kinase (AMPK), mice were intraperitoneally injected with compound C (CC). To overexpress pH domain and leucine-rich repeat protein phosphatase 1 (PHLPP1), lentiviral vectors were used.

**Results:**

The miR-299a-5p expression in the spinal cord was dramatically reduced by SCI stimulation. The miR-299a-5p agomir prevents, while the miR-299a-5p antagomir exacerbates inflammation, oxidative stress, and SCI in mice. Mechanistically, we found that miR-299a-5p directly inhibited PHLPP1 and subsequently activated AMPK pathway. The PHLPP1 overexpression of AMPK inhibition with either genetic or pharmacologic methods dramatically abolished the miR-299a-5p agomir-mediated protective effects against SCI.

**Conclusion:**

miR-299a-5p protects against spinal cord injury through activating AMPK pathway.

## 1. Introduction

Spinal cord injury (SCI) is a devastating central nervous system damage that can cause motor, sensory, and autonomic dysfunction, while no effective therapies are currently available. Multiple mechanisms are implicated in the pathogenesis of SCI, including inflammation and oxidative stress. Upon SCI, microglia cells, the resident macrophages in the spinal cord, are activated and then produce massive proinflammatory cytokines, resulting in inflammatory damage and leukocyte infiltration [1, 2]. In addition, the blood-spinal cord barrier (BSCB) is severely disrupted immediately after SCI and lasting for at least 28 days, which also exacerbates tissue edema, leukocyte extravasation, and inflammation [3]. Moreover, the level of reactive oxygen species (ROS) in the spinal cord is elevated by SCI and contributes to the progression of SCI by triggering peroxidation of lipid, protein, and nucleic acid [2, 4]. Based on these contexts, it is desirable to prevent SCI through targeting inflammation and oxidative stress.

AMP-activated protein kinase (AMPK) is a serine/threonine protein kinase and mainly involves in regulating cellular energy homeostasis. In addition, it also participates in some other biological processes, such as inflammation and oxidative stress [5–8]. Emerging studies have demonstrated that AMPK activation effectively inhibits the phosphorylation and activation of nuclear factor-*κ*B (NF-*κ*B), thereby preventing SCI-induced inflammation and motor dysfunction [9]. Besides, AMPK activation could also suppress the activation of nucleotide-binding domain-like receptor protein 3 (NLRP3) inflammasome, an intracellular multiprotein complex to promote the processing, maturation, and secretion of multiple proinflammatory cytokines [10, 11]. Transcriptional factor nuclear factor E2-related factor 2 (NRF2) plays an important role in maintaining cellular redox homeostasis through increasing the expression of antioxidant enzymes, including NAD (P)H: quinone oxidoreductase 1 (NQO1), superoxide dismutase 2 (SOD2), and catalase (CAT) [12, 13]. Hu et al. recently identified that AMPK activation dramatically elevated the NRF2 expression and eventually reduced SCI-induced oxidative stress and functional impairment [14]. Therefore, finding novel regulators of AMPK, especially endogenous activators, is critical to treat SCI.

MicroRNAs (miRs) are endogenous small noncoding RNAs to regulate gene expressions through binding to the 3′-untranslational region (3′-UTR) of targeted messenger RNAs, and they are shown to be responsible for the progression of SCI [15–17]. Most studies about miR-299a-5p mainly focus on its role in regulating tumor growth and chemotherapy sensitivity; however, its function and molecular basis during SCI remain unclear [18]. Sun et al. previously revealed a correlation between miR-299a-5p and sepsis-related inflammation and acute kidney injury [19]. And the predicted targets of miR-299a-5p are known to affect inflammation and oxidative stress [20]. Based on these results, we hypothesize that miR-299a-5p may be implicated in the pathogenesis of inflammation, oxidative stress, and SCI in mice.

## 2. Materials and Methods

### 2.1. Chemicals

Compound C (CC, #S7840) was purchased from Selleck Chemicals (Houston, TX, USA). Evans blue dye (#E2129), 2′,7′-dichlorofluorescin diacetate (DCFH-DA, #D6883), and lucigenin (#M8010) were purchased from Sigma-Aldrich (St. Louis, MO, USA). Amplex™ Red Hydrogen Peroxide/Peroxidase Assay Kit (#A22188) and NE-PER™ Nuclear and Cytoplasmic Extraction Reagents (#78833) were purchased from Thermo Fisher Scientific (Waltham, MA, USA). Commercial kits to detect myeloperoxidase (MPO, #ab155458), malondialdehyde (MDA, #ab118970), 3-nitrotyrosine (3-NT, #ab116691), 8-hydroxy-2-deoxyguanosine (8-OHdG, #ab201734), total SOD (#ab65354) activity, total antioxidant capacity (#ab65329), and capase-1 (casp1, #ab273268) activity were purchased from Abcam (Cambridge, UK). Interleukin-6 (IL-6, #M6000B), tumor necrosis factor-*α* (TNF-*α*, #MTA00B), IL-1*β* (#MLB00C), and IL-18 (#7625) ELISA kits were purchased from R&D Systems (Minneapolis, MN, USA). TransAM® NF-*κ*B p65 assay kit (#40096) and TransAM NRF2 assay kit (#50296) were obtained from Active Motif (Carlsbad, CA, USA). The following primary antibodies were purchased from Cell Signaling Technology (Danvers, MA, USA): anti-phospho-NF-*κ*B p65 (p-p65, #3033), anti-total p65 (t-p65, #8242), anti-p-AMPK (#2535), and anti-t-AMPK (#5831). Anti-NLRP3 (#ab263899), anti-apoptosis-associated speck-like protein (ASC, #ab47092), anti-glyceraldehyde-3-phosphate dehydrogenase (GAPDH, #ab8245), and anti-NRF2 (#ab62352) were purchased from Abcam. Anti-casp1 p10 (#sc-56036) was purchased from Santa Cruz Biotechnology (Dallas, Texas, USA), while anti-pH domain and leucine-rich repeat protein phosphatase 1 (PHLPP1, #67640-1-Ig) were purchased from Proteintech Group, Inc. (Rosemont, IL, USA). The micrON mmu-miR-299a-5p agomir (#miR40000377-4-5), agomir negative control (agomir-NC, #miR4N0000001-4-5), micrOFF mmu-miR-299a-5p antagomir (#miR30000377-4-5), and antagomir-NC (#miR3N0000001-4-5) were purchased from Guangzhou RiboBio Co., Ltd. (Guangzhou, China). Lentivirus carrying the full-length mouse PHLPP1 (NM_133821.3) or the scramble control (Ctrl) was synthetized by Shanghai Genechem Co., Ltd. (Shanghai, China).

### 2.2. Animals

Twelve-week-old male C57BL/6 mice were group-housed at five per cage with free access to food and water in a 12/12 h light-dark cycle at 22-25°C, and the feeding conditions were kept in a specific pathogen-free barrier system at Wuhan University. Animal experimental procedures were approved by the Ethics Committee of Zhongnan Hospital of Wuhan University (Approval no. ZN2022099). The SCI mouse model was established according to previous studies [2, 21]. Briefly, mice were anesthetized by isoflurane and received a laminectomy with the spinal cord exposed at the T8 vertebral level. Then, the vertebral column was stabilized and subjected to a 60 kdyn contusion using the Infinite Horizons Impactor (Precision Systems and Instrumentation, Fairfax Station, VA, USA). The successful surgery was confirmed by the trembled body, stretched and turned legs, and dropped tail. Penicillin sodium solution was administered once daily for 3 consecutive days postsurgery for disinfection, and 2 mL sterile saline was subcutaneously injected to help rehydration. After SCI, bladders were manually voided twice daily until bladder function was restored. In the sham-operated groups, mice were exposed to a laminectomy at the T8 vertebral level without injury. To overexpress or inhibit miR-299a-5p, mice were intrathecally injected with the agomir, antagomir, or matched NC of miR-299a-5p at a dose of 0.5 nmol per mouse according to previous studies [17, 22]. To inhibit AMPK, 20 mg/kg CC was intraperitoneally injected once two days from 1 week before SCI surgery [10]. To overexpress PHLPP1, SCI mice were injected with 2 *μ*L lentivirus (1 × 10^8^ TU/mL) at rostral and caudal sites 3 mm from the lesion epicenter with approximately 0.5 mm in depth [23]. All mice were sacrificed 7 days after SCI surgery for molecular detection except special annotations.

### 2.3. Behavioral Analysis and Sensitivity to Mechanical and Thermal Stimulation

Basso Mouse Scale (BMS) score was calculated before or at 1, 3, 7, 14, and 28 days after SCI to evaluate hindlimb function as previously described [3, 21]. In brief, mice were placed in an open field, and the posterior ankle joint mobility, trunk position and stability, coordination of front and rear limbs, paw posture, toe clearance, and tail position were recorded by two observers blind to the experimental condition, from 0 (no ankle movement) to 9 (normal gait) for scoring. Mechanical allodynia and thermal sensitivity were measured according to a previous study at 28 days after SCI [2].

### 2.4. Evaluation of BSCB Permeability

BSCB permeability was evaluated by measuring the extravasation of Evans blue dye at 28 days after SCI as previously described [3]. Briefly, mice were intravenously injected with 2% Evans blue dye and allowed circulating for 3 h. Next, the spinal cord lesion was collected, homogenized, and incubated in 50% trichloroacetic acid solution at 60°C for 24 h. After that, the supernatants were collected and detected at 620 nm excitation and 680 emission using a spectrophotometer.

### 2.5. Quantitative Real-Time PCR

Total RNA was extracted using TRIzol Reagent (Thermo Fisher Scientific), and then 2 *μ*g total RNA was reversely transcribed to cDNA using the first strand cDNA synthesis kit (Roche, Base, Switzerland) according to previous studies [24–26]. Quantitative real-time PCR was then done using the SYBR Green Master mix (Roche) on the LightCycler 480 system (Roche), and GAPDH as well as U6 was selected as the internal controls for mRNA or miRNA, respectively. Relative mRNA levels were calculated using 2^-∆∆Ct^ formula. The thermocycling conditions were provided as below: 95°C for 30 sec, 40 cycles at 95°C for 5 sec, and 60°C for 30 sec. The primer sequences were provided as below: miR-299a-5p, forward, 5′-ACACTCCAGCTGGGTGGTTTACCGTCCCAC-3′ and reverse, 5′-CTCAACTGGTGTCGTGGAGTCGGCAATTCAGTTGAGATGTATGT-3′; U6, forward, 5′-CTCGCTTCGGCAGCACA-3′ and reverse, 5′-AACGCTTCACGAATTTGCGT-3′; NQO-1, forward, 5′-AGGATGGGAGGTACTCGAATC-3′ and reverse, 5′-AGGCGTCCTTCCTTATATGCTA-3′; SOD2, forward, 5′-CAGACCTGCCTTACGACTATGG-3′ and reverse, 5′-CTCGGTGGCGTTGAGATTGTT-3′; CAT, forward, 5′-AGCGACCAGATGAAGCAGTG-3′ and reverse, 5′-TCCGCTCTCTGTCAAAGTGTG-3′; PHLPP1, forward, 5′-AGGGTCCCGGAGACGATAAG-3′ and reverse, 5′-AGGGCGGAGATGTCTTTTGC-3′; GAPDH, forward, 5′-AGGTCGGTGTGAACGGATTTG-3′ and reverse, 5′-TGTAGACCATGTAGTTGAGGTCA-3′.

### 2.6. Western Blot

Total proteins were extracted using RIPA lysis buffer containing protease/phosphatase inhibitor cocktail, and the concentrations were quantified by Pierce™ BCA Protein Assay kit as previously described [27–30]. Then, the proteins were separated by 10% SDS-PAGE, transferred onto PVDF membranes, and incubated with the primary antibodies at 4°C overnight. On the second day, the membranes were incubated with horseradish peroxidase-conjugated secondary antibodies and then visualized using the electrochemiluminescence detection system on a ChemiDoc XRS+ Image System (Bio-Rad; Hercules, California, USA). Next, the images were analyzed using Image Lab software (Version 6.0) and normalized to match total proteins or GAPDH.

### 2.7. Analysis of ROS Level

The level of intracellular ROS level was measured using DCFH-DA probe as previously described by us and the others [25, 31, 32]. Briefly, the spinal cord was homogenized and incubated with 50 *μ*mol/L DCFH-DA solution at 37°C for 30 min, and then the fluorescent intensities were determined at 504/524 nm to evaluate intracellular ROS level. To evaluate the level of hydrogen peroxide, the spinal cord was homogenized and reacted with the Amplex™ Red reagent according to the manufacturer's instructions, with the absorbance measured at 560 nm. Superoxide anion was quantified by incubating with 5 mmol/L lucigenin at 37°C for 10 min, and the luminescence intensity was measured at 30 sec intervals for 3-5 min.

### 2.8. Biochemical Analysis

The activities of MPO, casp1 activity, total SOD, and TAOC were determined by commercial kits according to the manufacturer's instructions. To evaluate NF-*κ*B and NRF2 transcriptional activities, nuclear extracts were prepared using the NE-PER™ Nuclear and Cytoplasmic Extraction Reagents and then incubated with the TransAM® NF-*κ*B p65 Kit or TransAM NRF2 Kit. In separated studies, the fresh spinal cord homogenates were exposed to enzyme-linked immunosorbent assay (ELISA) detection of the IL-6, TNF-*α*, IL-1*β*, and IL-18 using commercial ELISA kits. The levels of MDA, 3-NT, and 8-OHdG were determined as previously described by us and the others [25, 31, 33].

### 2.9. Luciferase Reporter Assay

The wild type and truncated 3′-UTR of PHLPP1 across the seed binding region (NM_133821.3) were obtained from GeneCopoeia (Rockville, MD, USA) and amplified using PCR, which were then cloned into the pGL3-Basic plasmid (Promega, Madison, Wisconsin, USA) downstream of the luciferase reporter gene. Next, these plasmids as well as pRL-TK plasmid were transfected into HEK293T cells with or without the miR-299a-5p agomir using Lipofectamine 3000 for 48 h. The luciferase activity was measured using the Dual-Luciferase Report Assay System (Promega) as we recently described, and changes in the ratio of firefly luciferase and Renilla luciferase were measured to evaluate the interaction between miR-299a-5p and PHLPP1 3′-UTR [25, 34, 35].

### 2.10. Statistical Analysis

Data are expressed as the mean ± standard deviation and analyzed using SPSS 23.0 software. Two-tailed unpaired Student's *t*-test was used to compare 2 groups, while one-way analysis of variance (ANOVA) followed by the Tukey posthoc test was conducted to compare the differences among 3 or more groups. Before one-way ANOVA analysis, Gaussian distribution was conducted using D'Agostino and Pearson omnibus normality test. *P* < 0.05 was considered statistically significant.

## 3. Results

### 3.1. The miR-299a-5p Agomir Prevents SCI in Mice

**Figure 1:**
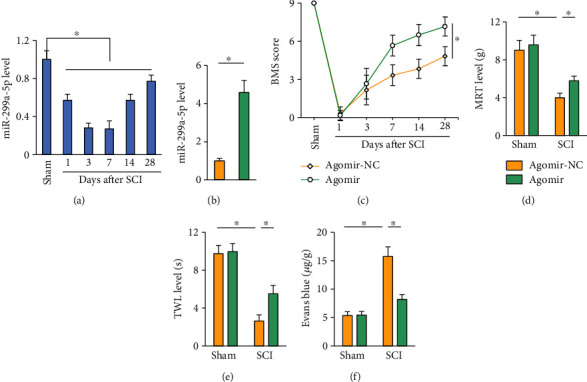
The miR-299a-5p agomir prevents SCI in mice. (a) The expression of miR-299a-5p in the spinal cord after SCI. (b) The expression of miR-299a-5p in mice treated with the miR-299a-5p agomir or agomir-NC. (c) BMS score. (d, e) Sensitivities to mechanical and thermal stimulation. (f) Extravasation of Evans blue dye. *n* = 6 for each groups. Data are expressed as the mean ± standard deviation, and *P* < 0.05 was considered statistically significant.

We first detect the alteration of miR-299a-5p level during SCI. As shown in Figure 1(a), the miR-299a-5p expression in the spinal cord was dramatically reduced by SCI stimulation but partially restored at 14 or 28 days after SCI. To investigate the function of miR-299a-5p, mice were treated with the miR-299a-5p agomir to overexpression its expression in the spinal cord (Figure 1(b)). BMS scoring data indicated that treatment with the miR-299a-5p agomir significantly prevented SCI-induced motor dysfunction in mice (Figure 1(c)). In addition, the hypersensitivity to mechanical and thermal stimulation in SCI mice was also improved by the miR-299a-5p agomir, as determined by the enhanced mechanical response threshold (MRT) and thermal withdrawal latency (TWL) (Figures 1(d) and 1(e)). BSCB disruption contributes to the progression of SCI, and accordingly, we identified a significant leakage of Evans blue dye in SCI mice, which could be alleviated by the miR-299a-5p agomir (Figure 1(f)). Collectively, we demonstrate that the miR-299a-5p agomir prevents SCI in mice.

### 3.2. The miR-299a-5p Antagomir Exacerbates SCI in Mice

**Figure 2:**
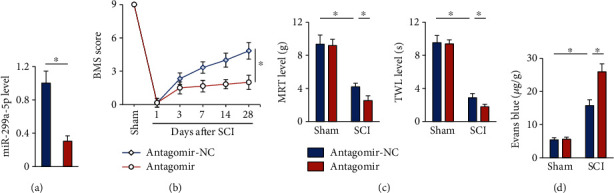
The miR-299a-5p antagomir exacerbates SCI in mice. (a) The expression of miR-299a-5p in mice treated with the miR-299a-5p antagomir or antagomir-NC. (b) BMS score. (c) Sensitivities to mechanical and thermal stimulation. (d) Extravasation of Evans blue dye. *n* = 6 for each groups. Data are expressed as the mean ± standard deviation, and *P* < 0.05 was considered statistically significant.

We then investigated whether downregulating the miR-299a-5p expression could further aggravated SCI in mice by treating mice with the miR-299a-5p antagomir (Figure 2(a)). As shown in Figure 2(b), the miR-299a-5p antagomir did not affect motor function under basal conditions but dramatically delayed the recovery of motor function in SCI mice. SCI-induced mechanical allodynia and thermal hypersensitivity were also exacerbated by the miR-299a-5p antagomir (Figure 2(c)). Moreover, treatment with the miR-299a-5p antagomir further disrupted BSCB integrity upon SCI surgery, as evidenced by the increased leakage of Evans blue dye (Figure 2(d)). Collectively, we demonstrate that the miR-299a-5p antagomir exacerbates SCI in mice.

### 3.3. The miR-299a-5p Agomir Inhibits SCI-Induced Inflammation and NLRP3 Inflammasome

**Figure 3:**
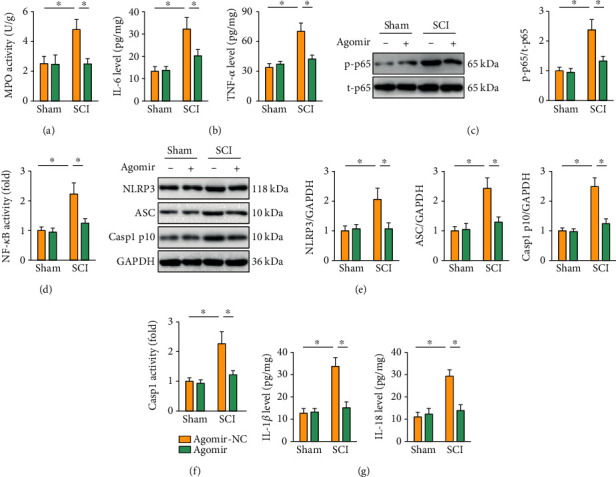
The miR-299a-5p agomir inhibits SCI-induced inflammation and NLRP3 inflammasome. (a) MPO activity in the spinal cord from mice treated with the miR-299a-5p agomir or agomir-NC. (b) IL-6 and TNF-*α* levels in the spinal cord. (c) Western blot images and quantification of p65 phosphorylation. (d) Relative NF-*κ*B transcriptional activity. (e) Western blot images and quantification of NLRP3, ASC, and casp1 p10. (f) Relative casp1 activity. (g) IL-1*β* and IL-18 levels in the spinal cord. *n* = 6 for each groups. Data are expressed as the mean ± standard deviation, and *P* < 0.05 was considered statistically significant.

Inflammation contributes to the progression of SCI, and we then evaluated whether it is involved in the protective role against SCI by the miR-299a-5p agomir. MPO activity, an index to determine neutrophils infiltration, was significantly increased in SCI mice but decreased in those treated with the miR-299a-5p agomir (Figure 3(a)). And the miR-299a-5p agomir also reduced IL-6 and TNF-*α* levels in the spinal cord from SCI mice (Figure 3(b)). NF-*κ*B is a central transcriptional factor to elicit the expression of multiple proinflammatory cytokines, and its activation accelerates the development of SCI [3, 36]. And we found that treatment with the miR-299a-5p agomir dramatically suppressed p65 phosphorylation and activation (Figures 3(c) and 3(d)). NLRP3 inflammasome is essential for the processing, maturation, and secretion of intracellular proinflammatory cytokines (e.g., IL-1*β* and IL-18) and participates SCI-induced inflammation and motor dysfunction [2, 37]. Intriguingly, the expression and activity of NLRP3 inflammasome components were evidently reduced in the miR-299a-5p agomir-treated SCI mice, as determined by the decreased NLRP3, ASC, and casp1 p10 expression and casp1 activity (Figures 3(e) and 3(f)). Accordingly, the levels of IL-1*β* and IL-18 in SCI mice were also reduced by the miR-299a-5p agomir (Figure 3(g)). Taken together, our findings reveal that the miR-299a-5p agomir inhibits SCI-induced inflammation and NLRP3 inflammasome.

### 3.4. The miR-299a-5p Agomir Suppresses SCI-Induced Oxidative Stress

**Figure 4:**
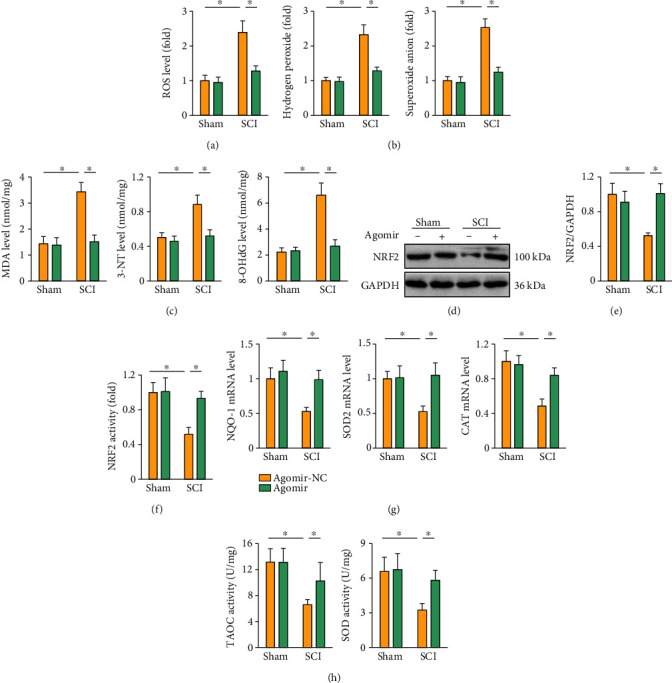
The miR-299a-5p agomir suppresses SCI-induced oxidative stress. (a) ROS level detected by DCFH-DA probe. (b) Quantification of hydrogen peroxide and superoxide anion in the spinal cord. (c) The levels of MDA, 3-NT, and 8-OHdG. (d)–(f) Relative levels of NRF2 protein and transcriptional activity. (g) The mRNA levels of NQO-1, SOD2, and CAT in the spinal cord. (h) Quantification of TAOC and total SOD activities. *n* = 6 for each groups. Data are expressed as the mean ± standard deviation, and *P* < 0.05 was considered statistically significant.

Oxidative stress is another feature and pathological factor of SCI. As expected, SCI surgery significantly increased the level of ROS in the spinal cord, as indicated by the elevated ROS, hydrogen peroxide, and superoxide anion levels, which were all reduced in the presence of the miR-299a-5p agomir (Figures 4(a) and 4(b)). Excessive ROS triggers peroxidation of lipid, protein, and nucleic acid, eventually leading neuron loss and motor dysfunction during SCI. As shown in Figure 4(c), treatment with the miR-299a-5p agomir significantly reduced the levels of MDA, 3-NT, and 8-OHdG. NRF2 plays critical roles in orchestrating the transcription of various antioxidant enzymes, and its suppression contributes to the progression of SCI. In line with the decreased oxidative stress, we found that the miR-299a-5p agomir dramatically restored the NRF2 expression in SCI mice (Figures 4(d) and 4(e)). Besides, NRF2 transcriptional activity was enhanced in the miR-299a-5p agomir-treated SCI mice, as further confirmed by the increased mRNA levels of downstream targets, including NQO-1, SOD2, and CAT (Figures 4(f) and 4(g)). Accordingly, SCI-induced suppression on TAOC and total SOD activity was significantly prevented by the miR-299a-5p agomir (Figure 4(h)). These data indicate that the miR-299a-5p agomir suppresses SCI-induced oxidative stress.

### 3.5. The miR-299a-5p Antagomir Exacerbates SCI-Induced Inflammation and Oxidative Stress

**Figure 5:**
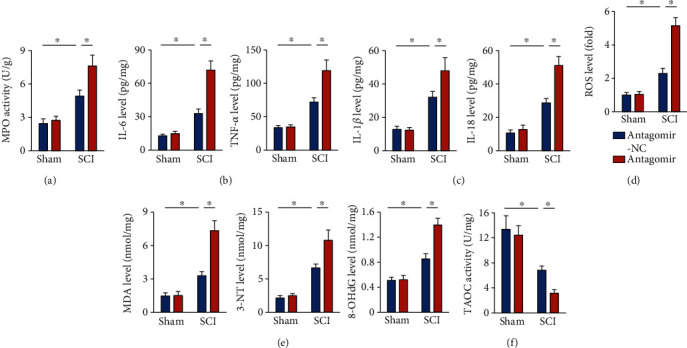
The miR-299a-5p antagomir exacerbates SCI-induced inflammation and oxidative stress. (a) MPO activity in the spinal cord from mice treated with the miR-299a-5p antagomir or antagomir-NC. (b) IL-6 and TNF-*α* levels in the spinal cord. (c) IL-1*β* and IL-18 levels in the spinal cord. (d) ROS level detected by DCFH-DA probe. (e) The levels of MDA, 3-NT, and 8-OHdG. (f) Quantification of TAOC activity. *n* = 6 for each groups. Data are expressed as the mean ± standard deviation, and *P* < 0.05 was considered statistically significant.

In contrast with the protective phenotypes in the miR-299a-5p agomir-treated SCI mice, we found that treatment with the miR-299a-5p antagomir significantly promoted neutrophil infiltration to the spinal cord upon SCI stimulation (Figure 5(a)). Accordingly, SCI-induced elevations of IL-6, TNF-*α*, IL-1*β*, and IL-18 levels were also amplified in the presence of the miR-299a-5p antagomir (Figures 5(b) and 5(c)). In addition, the miR-299a-5p antagomir-treated mice also exhibited increased oxidative stress under SCI stress, as evidenced by the increased ROS, MDA, 3-NT, and 8-OHdG productions and decreased TAOC level (Figures 5(d)–5(f)). Yet, no difference of survival rate was observed between groups (data not shown). Taken together, we suppose that the miR-299a-5p antagomir exacerbates SCI-induced inflammation and oxidative stress.

### 3.6. The miR-299a-5p Agomir Protects against SCI in Mice through Activating AMPK

**Figure 6:**
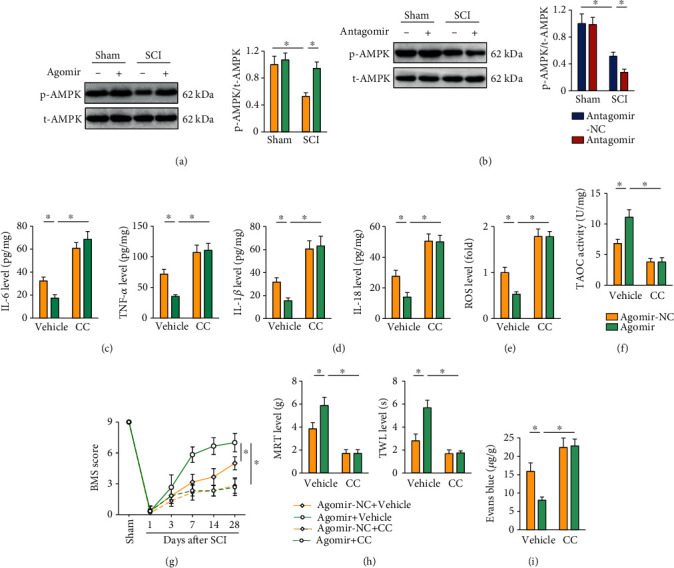
The miR-299a-5p agomir protects against SCI in mice through activating AMPK. (a, b) Western blot images and quantification of AMPK phosphorylation in mice treated with the miR-299a-5p agomir or antagomir. (c) IL-6 and TNF-*α* levels in the spinal cord. (d) IL-1*β* and IL-18 levels in the spinal cord. (e) ROS level detected by DCFH-DA probe. (f) Quantification of TAOC activity. *n* = 6 for each groups. (g) BMS score. (h) Sensitivities to mechanical and thermal stimulation. (i) Extravasation of Evans blue dye. *n* = 6 for each groups. Data are expressed as the mean ± standard deviation, and *P* < 0.05 was considered statistically significant.

Given the multifunctional role of AMPK and its involvement in the pathogenesis of SCI, we then tried to investigate whether the miR-299a-5p agomir prevented SCI through activating AMPK. As shown in Figures 6(a) and 6(b), the miR-299a-5p agomir increased, while the miR-299a-5p antagomir decreased AMPK phosphorylation in SCI mice. To further validate the necessity of AMPK, SCI mice were treated with CC to inhibit AMPK. Intriguingly, CC treatment completely blocked the inhibitory role of the miR-299a-5p agomir on SCI-induced inflammation, as evidenced by the increased IL-6, TNF-*α*, IL-1*β*, and IL-18 levels (Figures 6(c) and 6(d)). In addition, the miR-299a-5p agomir failed to suppress oxidative stress in SCI mice in the presence with CC (Figures 6(e) and 6(f)). In line with the molecular alterations, CC treatment abrogated the miR-299a-5p agomir-mediated protections against SCI-induced motor dysfunction, mechanical allodynia, and thermal hypersensitivity (Figures 6(g) and 6(h)). And BSCB disruption was further exacerbated in the miR-299a-5p agomir-treated SCI mice by CC, as confirmed by the increased Evans blue leakage to spinal cord (Figure 6(i)). Our findings suggest that the miR-299a-5p agomir protects against SCI in mice through activating AMPK.

### 3.7. The miR-299a-5p Agomir Activates AMPK through Downregulating PHLPP1

**Figure 7:**
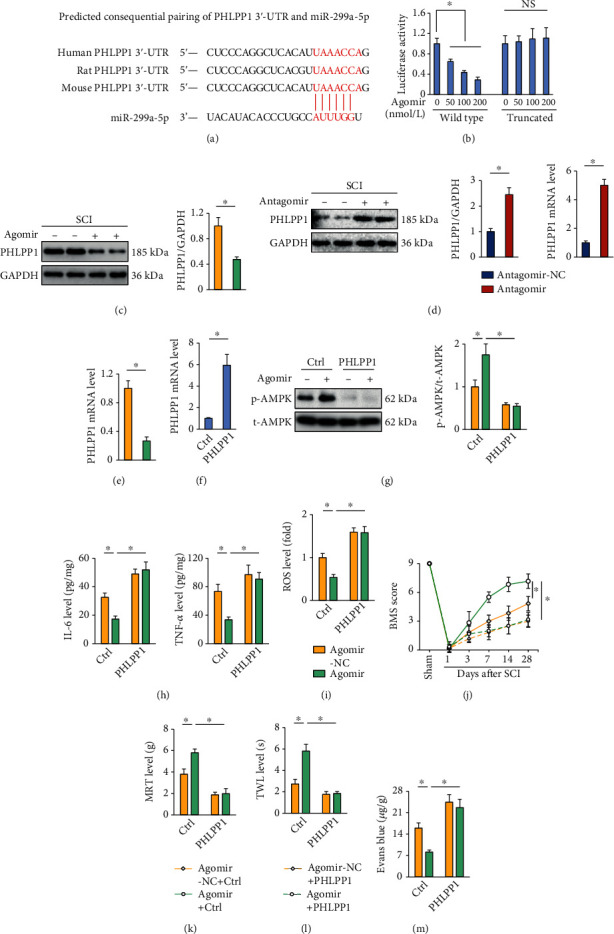
The miR-299a-5p agomir activates AMPK through downregulating PHLPP1. (a) The PHLPP1 3′-UTR contains a conserved binding site for miR-299a-5p. (b) The luciferase assay indicated that miR-299a-5p directly bound to the PHLPP1 3′-UTR. (c)–(e) Quantification of PHLPP1 protein and mRNA levels in SCI mice treated with the agomir, antagomir, or matched NC of miR-299a-5p. (f) The mRNA levels of PHLPP1 in mice with or without PHLPP1 overexpression. (g) Western blot images and quantification of AMPK phosphorylation in the miR-299a-5p agomir-treated mice with or without PHLPP1 overexpression. (h) IL-6 and TNF-*α* levels in the spinal cord. (i) ROS level detected by DCFH-DA probe. (j) BMS score. (k, l) Sensitivities to mechanical and thermal stimulation. (m) Extravasation of Evans blue dye. *n* = 6 for each groups. Data are expressed as the mean ± standard deviation, and *P* < 0.05 was considered statistically significant.

Finally, we explored the possible mechanism through which the miR-299a-5p agomir activated AMPK. Using the online TargetScan software, PHLPP1 was selected for further investigation due to its role in dephosphorylating AMPK and tissue injury [38, 39]. As shown in Figure 7(a), a conserved binding site was found in the PHLPP1 3′-UTR. And results using luciferase reporter assay further validate the directly interaction between miR-299a-5p and PHLPP1 3′-UTR (Figure 7(b)). In addition, the protein and mRNA levels of PHLPP1 in SCI mice were decreased by the miR-299a-5p agomir, while increased by the miR-299a-5p antagomir (Figures 7(c)–7(e)). To validate the necessity of PHLPP1 in regulating AMPK by miR-299a-5p, mice were injected with lentiviral vectors to overexpress PHLPP1 in vivo, and the efficiency was presented in Figure 7(f). As shown in Figure 7(g), the PHLPP1 overexpression completely abrogated the miR-299a-5p agomir-mediated AMPK activation in SCI mice. And the antiinflammatory and antioxidant capacities of the miR-299a-5p agomir were blocked in PHLPP1-overexpressed SCI mice (Figures 7(h) and 7(i)). Accordingly, the PHLPP1 overexpression abolished the miR-299a-5p agomir-mediated protective effects against SCI-induced motor dysfunction, mechanical allodynia, and thermal hypersensitivity (Figures 7(j)–7(l)). And BSCB disruption was further exacerbated in the miR-299a-5p agomir-treated SCI mice by the PHLPP1 overexpression (Figure 7(m)). In general, we prove that the miR-299a-5p agomir activates AMPK through downregulating PHLPP1.

## 4. Discussion

SCI is a devastating neurotrauma with severe and insufferable sequelae, such as motor deficits, neuropathic pain, and hypersensitivity. The present study found that the miR-299a-5p expression was downregulated during SCI, and that the miR-299a-5p agomir dramatically prevented SCI-induced inflammation, oxidative stress, and motor and sensory dysfunction. Conversely, treatment with the miR-299a-5p antagomir further exacerbated SCI in mice. Mechanistically, we observed that the miR-299a-5p agomir activated, while the miR-299a-5p antagomir inhibited AMPK pathway in SCI mice, and that AMPK inhibitor completely blocked the beneficial effects of the miR-299a-5p agomir in vivo. Further findings identified a conserved binding site of miR-299a-5p in PHLPP1 3′-UTR, and treatment with the miR-299a-5p agomir significantly decreased the PHLPP1 expression in SCI mice. Yet, the PHLPP1 overexpression blocked AMPK activation by the miR-299a-5p agomir in SCI mice, accompanied by an increased inflammation and oxidative stress. To the best of our knowledge, this is the first study about the pathophysiological role and molecular basis of miR-299a-5p during SCI progression.

Inflammation, manifested as extensive microglia cell activation and infiltrations of leukocytes, is a key feature and pathogenic factor of SCI [1]. Upon SCI, microglia cells are activated to synthetize multiple proinflammatory cytokines, which in turn recruit the infiltration of peripheral immune cells to the lesion and further amplify the inflammatory response [40]. The BSCB plays critical roles in controlling the movement of molecules, liquids, or cells between blood vessels and spinal cord, and its structural and functional integrities are required for the microenvironment homeostasis of the spinal cord. However, BSCB breakdown occurs as early as 5 min after SCI, accompanied by the infiltration of neutrophils and macrophages to the spinal cord [41]. The present study demonstrated that treatment with the miR-299a-5p agomir could prevent SCI-induced BSCB disruption and tissue inflammation in mice. NLRP3 inflammasome is essential for the processing and maturation of proinflammatory cytokines, and its activation contributes to SCI progression. Previous studies have shown that NLRP3 inactivation dramatically protects against motor dysfunction and sensory hypersensitivity in SCI mice [2, 4]. Consistently, we herein observed that SCI-induced activation of NLRP3 inflammasome was blunted by the miR-299a-5p agomir, accompanied by reduced IL-1*β* and IL-18 expressions in the spinal cord. Apart from inflammation, excessive ROS functions as another contributor of SCI. Findings from us and other laboratories have shown that the endogenous antioxidant capacities were suppressed by SCI surgery, followed by uncontrolled ROS generation [2, 3]. In addition, neurons in the spinal cord are especially vulnerable to free radicals due to the negligible regenerative capacities. Moreover, ROS overproduction also triggers the dissociation of thioredoxin interacting protein from thioredoxin, which subsequently interacts with and activates NLRP3 inflammasome [42]. Our findings revealed that SCI-induced inflammation and oxidative stress were dramatically reduced by the miR-299a-5p agomir.

miR-299a-5p is identified as a tumor suppressor in various human cancers, including breast cancer, hepatocellular cancer, thyroid cancer, and colorectal cancer [18, 43]. Yet, very few studies have been down in the context of noncancer diseases. Huang et al. previously demonstrated that miR-299a-5p was downregulated in human islets and *β*-cell under glucolipotoxic conditions, and that miR-299a-5p inhibition promoted *β*-cell apoptosis and impaired *β*-cell function in glucolipotoxic settings [44]. Herein, we found that the miR-299a-5p expression was decreased in the spinal cord upon SCI stimulation. The miR-299a-5p agomir alleviated, while the miR-299a-5p antagomir further exacerbated inflammation, oxidative stress, motor dysfunction, and sensory hypersensitivity in SCI mice. Mechanistically, we identified PHLPP1 as a direct target of miR-299a-5p in regulating AMPK and SCI. PHLPP1 is a novel serine/threonine protein phosphatase and directly dephosphorylates many downstream kinases, such as AKT and STAT1 [39, 45]. Behera et al. previously demonstrated that PHLPP1 also interacted with and directly dephosphorylated AMPK Thr172 in myoblasts without influencing its conventional upstream kinase [38]. Consistently, Balamurugan et al. recently also found that the overexpression of PHLPP1 significantly reduced the phosphorylation of AMPK Thr172 [46]. PHLPP1 is traditionally identified to participate in the pathogenesis of various human tumors; however, emerging studies reveal that it also plays critical roles in regulating inflammation, oxidative stress, and tissue injury. Wen et al. previously demonstrated that PHLPP1 deletion protected intestinal epithelial cells against inflammation-induced apoptosis and improved colitis in mice [47]. And PHLPP1 deficiency also reduced inflammation and prevented cardiomyocyte death and cardiac dysfunction [48]. Consistently, we found that PHLPP1 suppression evidently blocked SCI-induced inflammation and NLRP3 inflammasome in mice. Additionally, Mathur et al. found that PHLPP1 silence enhanced NRF2 expression and nuclear localization, thereby alleviating high glucose-induced oxidative stress and apoptosis during diabetic nephropathy [49]. And results from Zhang et al. also indicated that PHLPP1 knockdown promoted the nuclear expression and transcriptional activity of NRF2, preventing oxidative stress and apoptosis in high glucose-treated retinal ganglion cells [50]. In line with these findings, we also found that PHLPP1 inhibition by the miR-299a-5p agomir significantly elevated the NRF2 expression and transcriptional activity in SCI mice, thereby reducing oxidative damage in SCI mice.

In summary, our study identifies an involvement of miR-299a-5p in SCI progression.

## Data Availability

Data supporting the findings of this work are available from the corresponding author upon reasonable request.

## References

[1] Silva N. A., Sousa N., Reis R. L., Salgado A. J. (2014). From basics to clinical: a comprehensive review on spinal cord injury. *Progress in Neurobiology*.

[2] Qian J., Zhu W., Lu M., Ni B., Yang J. (2017). D-*β*-Hydroxybutyrate promotes functional recovery and relieves pain hypersensitivity in mice with spinal cord injury. *British Journal of Pharmacology*.

[3] Ge X., Tang P., Rong Y. (2021). Exosomal miR-155 from M1-polarized macrophages promotes EndoMT and impairs mitochondrial function via activating NF-*κ*B signaling pathway in vascular endothelial cells after traumatic spinal cord injury. *Redox Biology*.

[4] Liu Z., Yao X., Sun B. (2021). Pretreatment with kaempferol attenuates microglia-mediate neuroinflammation by inhibiting MAPKs-NF-*κ*B signaling pathway and pyroptosis after secondary spinal cord injury. *Free Radical Biology & Medicine*.

[5] Hu C., Zhang X., Wei W. (2019). Matrine attenuates oxidative stress and cardiomyocyte apoptosis in doxorubicin-induced cardiotoxicity _via_ maintaining AMPK _*α*_ /UCP2 pathway. *Acta Pharmaceutica Sinica B*.

[6] Yang L., Li X., Jiang A. (2020). Metformin alleviates lead-induced mitochondrial fragmentation via AMPK/Nrf2 activation in SH-SY5Y cells. *Redox Biology*.

[7] Zhang T., Liu J., Shen S., Tong Q., Ma X., Lin L. (2020). SIRT3 promotes lipophagy and chaperon-mediated autophagy to protect hepatocytes against lipotoxicity. *Cell Death and Differentiation*.

[8] Zhang X., Ma Z. G., Yuan Y. P. (2018). Rosmarinic acid attenuates cardiac fibrosis following long-term pressure overload via AMPK*α*/Smad3 signaling. *Cell Death & Disease*.

[9] Jiang X., Shen Z., Chen J. (2020). Irisin protects against motor dysfunction of rats with spinal cord injury via adenosine 5'-monophosphate (AMP)-activated protein kinase-nuclear factor kappa-B pathway. *Frontiers in Pharmacology*.

[10] Jiang W. L., Zhao K. C., Yuan W. (2020). MicroRNA-31-5p exacerbates lipopolysaccharide-induced acute lung injury via inactivating Cab39/AMPK*α*Pathway. *Oxidative Medicine and Cellular Longevity*.

[11] Wan P., Su W., Zhang Y. (2020). LncRNA H19 initiates microglial pyroptosis and neuronal death in retinal ischemia/reperfusion injury. *Cell Death and Differentiation*.

[12] Gao Q., Zhang G., Zheng Y. (2020). SLC27A5 deficiency activates NRF2/TXNRD1 pathway by increased lipid peroxidation in HCC. *Cell Death and Differentiation*.

[13] Warpsinski G., Smith M. J., Srivastava S. (2020). Nrf2-regulated redox signaling in brain endothelial cells adapted to physiological oxygen levels: consequences for sulforaphane mediated protection against hypoxia-reoxygenation. *Redox Biology*.

[14] Hu H., Xia N., Lin J. (2021). Zinc regulates glucose metabolism of the spinal cord and neurons and promotes functional recovery after spinal cord injury through the AMPK signaling pathway. *Oxidative Medicine and Cellular Longevity*.

[15] Qiao J., Zhao J., Chang S. (2020). MicroRNA-153 improves the neurogenesis of neural stem cells and enhances the cognitive ability of aged mice through the notch signaling pathway. *Cell Death and Differentiation*.

[16] Lv L. L., Feng Y., Wu M. (2020). Exosomal miRNA-19b-3p of tubular epithelial cells promotes M1 macrophage activation in kidney injury. *Cell Death and Differentiation*.

[17] Ni S., Luo Z., Jiang L. (2019). UTX/KDM6A deletion promotes recovery of spinal cord injury by epigenetically regulating vascular regeneration. *Molecular Therapy*.

[18] Wang Z., He L., Sun W. (2018). miRNA-299-5p regulates estrogen receptor alpha and inhibits migration and invasion of papillary thyroid cancer cell. *Cancer Management and Research*.

[19] Sun J., Cai X., Shen J., Jin G., Xie Q. (2020). Correlation between single nucleotide polymorphisms at the 3′-UTR of theNFKB1gene and Acute kidney injury in sepsis. *Genetic Testing and Molecular Biomarkers*.

[20] Padgett K. A., Lan R. Y., Leung P. C. (2009). Primary biliary cirrhosis is associated with altered hepatic microRNA expression. *Journal of Autoimmunity*.

[21] Liu H., Zhang J., Xu X. (2021). SARM1 promotes neuroinflammation and inhibits neural regeneration after spinal cord injury through NF-*κ*B signaling. *Theranostics*.

[22] Sabirzhanov B., Matyas J., Coll-Miro M. (2019). Inhibition of microRNA-711 limits angiopoietin-1 and Akt changes, tissue damage, and motor dysfunction after contusive spinal cord injury in mice. *Cell Death & Disease*.

[23] Patel M., Li Y., Anderson J. (2021). Gsx1 promotes locomotor functional recovery after spinal cord injury. *Molecular Therapy*.

[24] Zhang X., Hu C., Yuan Y. P. (2021). Endothelial ERG alleviates cardiac fibrosis via blocking endothelin-1-dependent paracrine mechanism. *Cell Biology and Toxicology*.

[25] Bao C., Zhang J., Xian S. Y., Chen F. (2021). MicroRNA-670-3p suppresses ferroptosis of human glioblastoma cells through targeting ACSL4. *Free Radical Research*.

[26] Zhang X., Hu C., Zhang N. (2021). Matrine attenuates pathological cardiac fibrosis via RPS5/p38 in mice. *Acta Pharmacologica Sinica*.

[27] Hu C., Zhang X., Zhang N. (2020). Osteocrin attenuates inflammation, oxidative stress, apoptosis, and cardiac dysfunction in doxorubicin-induced cardiotoxicity. *Clinical and Translational Medicine*.

[28] Zhan W., Liao X., Chen Z. (2020). LINC00858 promotes colorectal cancer by sponging miR-4766-5p to regulate PAK2. *Cell Biology and Toxicology*.

[29] Zhu Y., Yang L., Xu J. (2020). Discovery of the anti-angiogenesis effect of eltrombopag in breast cancer through targeting of HuR protein. *Acta Pharmaceutica Sinica B*.

[30] Zhang X., Hu C., Yuan X. P. (2021). Osteocrin, a novel myokine, prevents diabetic cardiomyopathy via restoring proteasomal activity. *Cell Death & Disease*.

[31] Zhang X., Hu C., Kong C. Y. (2020). FNDC5 alleviates oxidative stress and cardiomyocyte apoptosis in doxorubicin-induced cardiotoxicity via activating AKT. *Cell Death and Differentiation*.

[32] Hu C., Zhang X., Song P. (2020). Meteorin-like protein attenuates doxorubicin-induced cardiotoxicity via activating cAMP/PKA/SIRT1 pathway. *Redox Biology*.

[33] Hu C., Zhang X., Hu M. (2022). Fibronectin type III domain-containing 5 improves aging-related cardiac dysfunction in mice. *Aging Cell*.

[34] Han X., Jiang H., Qi J. (2020). Novel lncRNA UPLA1 mediates tumorigenesis and prognosis in lung adenocarcinoma. *Cell Death & Disease*.

[35] Valente T., Dentesano G., Ezquerra M. (2020). CCAAT/enhancer binding protein *δ* is a transcriptional repressor of *α*-synuclein. *Cell Death and Differentiation*.

[36] Sanchez-Lopez E., Ghia E. M., Antonucci L. (2020). NF-*κ*B-p62-NRF2 survival signaling is associated with high ROR1 expression in chronic lymphocytic leukemia. *Cell Death and Differentiation*.

[37] Zeng C., Duan F., Hu J. (2020). NLRP3 inflammasome-mediated pyroptosis contributes to the pathogenesis of non- ischemic dilated cardiomyopathy. *Redox Biology*.

[38] Behera S., Kapadia B., Kain V. (2018). ERK1/2 activated PHLPP1 induces skeletal muscle ER stress through the inhibition of a novel substrate AMPK. *Biochimica et Biophysica Acta - Molecular Basis of Disease*.

[39] Katsenelson K. C., Stender J. D., Kawashima A. T. (2019). PHLPP1 counter-regulates STAT1-mediated inflammatory signaling. *eLife*.

[40] Liu W., Tang P., Wang J. (2021). Extracellular vesicles derived from melatonin-preconditioned mesenchymal stem cells containing USP29 repair traumatic spinal cord injury by stabilizing NRF2. *Journal of Pineal Research*.

[41] Horner P. J., Popovich P. G., Mullin B. B., Stokes B. T. (1996). A quantitative spatial analysis of the blood-spinal cord barrier: II. Permeability after intraspinal fetal transplantation. *Experimental Neurology*.

[42] Dai X., Liao R., Liu C. (2021). Epigenetic regulation of TXNIP-mediated oxidative stress and NLRP3 inflammasome activation contributes to SAHH inhibition-aggravated diabetic nephropathy. *Redox Biology*.

[43] Shevde L. A., Metge B. J., Mitra A. (2010). Spheroid-forming subpopulation of breast cancer cells demonstrates vasculogenic mimicry via hsa-miR-299-5p regulated de novo expression of osteopontin. *Journal of Cellular and Molecular Medicine*.

[44] Huang Q., You W., Li Y. (2018). Glucolipotoxicity-inhibitedmiR-299-5pregulates pancreatic *β*-cell function and survival. *Diabetes*.

[45] Lupse B., Annamalai K., Ibrahim H. (2021). Inhibition of PHLPP1/2 phosphatases rescues pancreatic *β*-cells in diabetes. *Cell Reports*.

[46] Keerthana B., Medishetti R., Kotha J. (2022). PHLPP1 promotes neutral lipid accumulation through AMPK/ChREBP-dependent lipid uptake and fatty acid synthesis pathways. *iScience*.

[47] Wen Y. A., Li X., Goretsky T., Weiss H. L., Barrett T. A., Gao T. (2015). Loss of PHLPP protects against colitis by inhibiting intestinal epithelial cell apoptosis. *Biochimica et Biophysica Acta*.

[48] Tan Y., Li T., Hu M. (2022). PHLPP1 deficiency ameliorates cardiomyocyte death and cardiac dysfunction through inhibiting Mcl-1 degradation. *Cellular Signalling*.

[49] Mathur A., Pandey V. K., Kakkar P. (2018). Activation of GSK3*β*/*β*-TrCP axis via PHLPP1 exacerbates Nrf2 degradation leading to impairment in cell survival pathway during diabetic nephropathy. *Free Radical Biology & Medicine*.

[50] Zhang X., Lu Y., He N., Wang F. (2020). Downregulation of PHLPP1 ameliorates high glucose-evoked injury in retinal ganglion cells by attenuating apoptosis and oxidative stress through enhancement of Nrf2 activation. *Experimental Cell Research*.

